# P-Glycoprotein Inhibitor Tariquidar Plays an Important Regulatory Role in Pigmentation in Larval Zebrafish

**DOI:** 10.3390/cells10030690

**Published:** 2021-03-20

**Authors:** Natalia Kasica, Piotr Jakubowski, Jerzy Kaleczyc

**Affiliations:** 1 Department of Animal Anatomy, Faculty of Veterinary Medicine, University of Warmia and Mazury in Olsztyn, Oczapowskiego 13 Street, Box 105J, 10-719 Olsztyn, Poland; jerzy.kaleczyc@uwm.edu.pl; 2 Department of Pharmacology and Toxicology, School of Medicine, University of Warmia and Mazury in Olsztyn, Warszawska 30 Street, 11-041 Olsztyn, Poland; piotr.jakubowski@uwm.edu.pl

**Keywords:** zebrafish, pigment cells, melanophores, iridophores, tyrosinase, dopachrome tautomerase, melanocyte inducing transcription factor, purine nucleoside phosphorylase 4a, P-glycoprotein inhibitor, tariquidar

## Abstract

Zebrafish has emerged as a powerful model in studies dealing with pigment development and pathobiology of pigment diseases. Due to its conserved pigment pattern with established genetic background, the zebrafish is used for screening of active compounds influencing melanophore, iridophore, and xanthophore development and differentiation. In our study, zebrafish embryos and larvae were used to investigate the influence of third-generation noncompetitive P-glycoprotein inhibitor, tariquidar (TQR), on pigmentation, including phenotype effects and changes in gene expression of chosen chromatophore differentiation markers. Five-day exposure to increasing TQR concentrations (1 µM, 10 µM, and 50 µM) resulted in a dose-dependent augmentation of the area covered with melanophores but a reduction in the area covered by iridophores. The observations were performed in three distinct regions—the eye, dorsal head, and tail. Moreover, TQR enhanced melanophore renewal after depigmentation caused by 0.2 mM 1-phenyl-2-thiourea (PTU) treatment. qPCR analysis performed in 56-h post-fertilization (hpf) embryos demonstrated differential expression patterns of genes related to pigment development and differentiation. The most substantial findings include those indicating that TQR had no significant influence on leukocyte tyrosine kinase, GTP cyclohydrolase 2, tyrosinase-related protein 1, and forkhead box D3, however, markedly upregulated tyrosinase, dopachrome tautomerase and melanocyte inducing transcription factor, and downregulated purine nucleoside phosphorylase 4a. The present study suggests that TQR is an agent with multidirectional properties toward pigment cell formation and distribution in the zebrafish larvae and therefore points to the involvement of P-glycoprotein in this process.

## 1. Introduction

The popularity of zebrafish as a model for studying vertebrate embryogenesis and development is constantly increasing. Recently, it emerged as a powerful and advantageous model in studies dealing with pigment development and diseases [1,2]. The optical transparency of zebrafish embryos and their rapid ex utero development enables observing cell fate from the moment of fertilization. Specific external pigmentation in the zebrafish owes its pattern to three types of pigment cells—black melanophores (synthesizing melanin), yellow xanthophores (containing pteridines), and silver iridophores (packed with purine crystals) [2,3]. In zebrafish, two distinct pigment patterning occurs during the life cycle. During embryogenesis, all those cells originate, as in other vertebrates, from two distinct sources. The first source is the neural crest cells whose derivatives are responsible for the dermis and epidermis patterning [4]. In adults, melanophores contribute to the longitudinal dark stripes, while xanthophores and iridophores form the yellowish, silver interstripes of the dermis and epidermis [5,6]. The second one is the optic cup, which contributes to the outermost layer of the retina, the retinal pigment epithelium (RPE) [6,7]. During the early larval stage, melanophores generate stripes at the edges of myotomes and cover the head and the yolk with characteristically arranged compositions. A few iridophores are present in a close neighborhood of melanophores, whereas xanthophores are scattered widely over the body. Based on the studies dealing with tyrosinase activity it can be assumed that pigment development starts from RPE around 21.5 h post fertilization (hpf) and is followed by melanophore formation within the dorsolateral skin, where first cells are visible around 24 hpf [8]. The xanthophores appear next, followed by the iridophores. First xanthophores become weakly visible at 48 hpf in the dorsal head and start to produce strong yellow pigment around 72 hpf, both within the eye and on the head and whole dorsolateral skin in the proximity to melanophores [9]. Iridophores became visible in the eye at 42 hpf and then slowly appear around 72 hpf in the neighborhood of other pigment cells along the body [9,10]. The whole pigment patterning is completed on the third day post fertilization (dpf). The development of the striped pattern in zebrafish depends on a variety of genes that regulate pigment cell fate, proliferation, survival, migration, and differentiation [2]. In this regard, zebrafish serves as an excellent model, because of the sequenced genome and the availability of plenty of pigment mutants with known genetic backgrounds [2]. To understand better pigment disorders in humans, the most valuable results obtained in zebrafish would be those dealing with pigment pattern variations and mutations observed in melanocytes, which represent pigment cells commonly found in all vertebrates. Importantly, the melanocyte development in zebrafish and mammals shares similarities between developmental pathways, including the formation of the melanoblast and regulation by common genes (*mitf*, *sox10*, *dct*, *tyr*) (reviewed in [11]). The pathogenesis of human disorders with melanocyte perturbation, such as vitiligo, piebaldism, Waardenburg syndrome, and melanoma is still attracting great attention of many researchers, but there are still some shortages in its complete comprehension. In the zebrafish genes involved in melanin biosynthesis (e.g., tyrosinase, dopachrome tautomerase, or tyrosinase-related protein 1) have been already identified and serve as markers of melanophore differentiation (reviewed in [2]). All of them are directly regulated by microphthalmia-associated transcription factor (mitf), the central agent in the melanocyte specification. In the case of iridophores, genetic studies have revealed that mitf also regulates their development [10,12]; however, the leukocyte receptor tyrosine kinase (ltk) plays a crucial role in this process [13]. Surprisingly, based on recent investigations purine nucleoside phosphorylase 4a (*pnp4a*) has been proposed as a new marker of early iridoblast development and differentiation [10,12].

There are plenty of compounds influencing melanogenesis in zebrafish. Among them, there can be found melanogenic inhibitors and stimulators. The most proven effects are demonstrated by 1-phenyl-2-thiourea (PTU), arbutin, kojic acid, and 2-mercaptobenzothiazole (inhibitors), and a-melanocyte stimulating hormone (a-MSH) (stimulator) [14]. However, other melanogenic regulatory compounds are broadly discussed [15]. All these factors regulate pigmentation by affecting tyrosinase activity; however, the variety of the reported target pathways is great and is reflected by many agents such as COX2, glucocorticoids, cholinesterase, JNK, IRAK, GABA receptor, EGFRK/ERB, and several other discussed in the literature [16].

During our studies on anti-epileptic properties of tariquidar (TQR; *N*-[2-[[[4-[2-(3,4-Dihydro-6,7-dimethoxy-2(1*H*)-isoquinolinyl)ethyl]phenyl]amino]carbonyl]-4,5-dimethoxyphenyl]-3-quinolinecarboxamide) (data not yet published), we observed unexpected side effect consisting in altered pigmentation. TQR is one of the P-glycoprotein (P-gp, Abcb1) drug pump inhibitors. So far, investigations dealing with TQR have been performed in humans and mice and have revealed its clear noncompetitive inhibitory properties toward P-gp [17]. Our preliminary experiment demonstrated a significant rise in the intensity of rhodamine B fluorescence in the brain of zebrafish larvae after their preincubation with TQR, suggesting that TQR influenced P-gp and stopped the rhodamine B efflux. To date, investigations dealing with TQR were aimed at its role in chemotherapy and as a factor increasing brain penetration of drugs [18,19]. However, the mechanism of Pg-p inhibition by TQR still requires better understanding and is continually under clinical trials. Pg-p is an evolutionary well-conserved ATP-binding cassette (ABC) efflux drug pump responsible for pumping out of cells xenobiotic substances. The P-gp expression has been found for the first time in cells of the human liver, pancreas, kidney, colon, and jejunum [20]. P-gp is also present in the capillary endothelial cells composing the blood–brain barrier [21,22,23]. More recently, P-gp (Abcb1) has been also identified in human and pig RPE [24,25,26], and Abcb5 protein, which is highly homologous to Abcb1, was found in melanoma cells and intact melanocytes [27]. In humans, P-gp is encoded by *abcb1*/*mdr1*gene [19]. Fischer et al. (2013) [28] have found in the zebrafish two genes, *abcb4* and *abcb5*, both of which are structurally similar to mammalian *abcb1*. These authors have also revealed that Abcb4, but not Abcb5, protein possesses functional properties similar to those of mammalian Abcb1. However, the tissue distribution of P-gp in zebrafish is still not fully recognized.

Therefore, the aim of the present study was to demonstrate, for the first time, the influence of third-generation noncompetitive P-gp inhibitor, TQR, on pigment cells, specifically melanophores and iridophores, in zebrafish, including the phenotype effects, and to analyze the expression of genes for chosen chromatophore differentiation markers.

## 2. Materials and Methods

### 2.1. Animals

The study was performed on wild-type Tuebingen (kindly gifted from the Nüsslein-Volhard Lab, Max-Planck-Institut für Entwicklungsbiologie in Tübingen, Germany) embryos and larvae aged from 4 hpf to 120 hpf. Adult fish were maintained in 8l tanks at 28 °C with a 14 h light: 10 h dark photoperiod and fed three times daily ad libitum with dry food and Artemia sp. nauplii. (Ocean Nutrition, Newark, CA, USA). The males and females were kept together in tanks. Spawning was made by transferring one male and one female to breeding tanks in the evening. The eggs were collected the next morning and transferred into Petri dishes with embryo solution (E3) containing 5 mM NaCl, 0.17 mM KCl, 0.33 mM CaCl_2_, and 0.33 mM MgSO_4_. Depending on the type of the experiment, all good quality 4 or 9 hpf embryos were distributed to experimental well plates. The individuals used in the study were anesthetized by placing them in a tricaine methanesulfonate (MS-222) (Sigma Aldrich, Munich, Germany) solution and euthanized by an overdose of MS-222, respectively.

### 2.2. Chemicals and Study Design

To investigate the mechanism of TQR (APExBIO, Houston, TX, USA) influence on pigmentation, three separate experiments were carried out. In all, each experimental group consisted of 15 individuals. The embryos of the control group were incubated in embryo solution (E3) with 0.1% dimethyl sulfoxide DMSO (Sigma Aldrich, Munich, Germany). TQR exposed embryos were assigned into three groups with increasing TQR concentration: 1 uM, 10 uM, and 50 uM. As a solvent for TQR, DMSO was used. At 20 hpf, the embryos were dechorionated with forceps. Replacement of mediums was performed daily. To evaluate how TQR impacts the pigment patterning from the moment of fertilization, the exposure was set in 6-well plates and lasted from 4 hpf until 120 hpf. The second aim was to determine the impact of TQR on pigment cell formation preceded by depigmentation by 0.2 mM PTU (Sigma Aldrich, Munich, Germany). For this purpose, following Choi et al. (2007) [14], 9-hpf embryos were exposed to 0.2 mM PTU. At 35 hpf, the embryos were washed and PTU was replaced by E3 or TQR. The phenotype was assessed at 65 hpf. Moreover, the effects of TQR exposure on iridophores were also evaluated. To exclude interferences between iridophores and melanophores and investigate the particular influence on chosen cell type, the 9-hpf embryos were exposed to the mixture of 0.2 mM PTU and increasing doses of TQR—1 µM, 10 µM, and 50 µM, respectively. The scoring (phenotype-based evaluation) was demonstrated at 120 hpf. To provide the standard maintenance conditions, the individuals were kept in the incubator with 28.5 °C and 14 h light:10 h dark photoperiod.

### 2.3. Phenotype-Based Evaluation

Observations of pigment patterning during embryogenesis were carried out daily at 30, 48, 72, 96, and 120 hpf, while photo documentation was performed at 30, 72, and 120 hpf. PTU-exposed embryos, depending on the type of the experiment, were evaluated at 35 hpf, 65 hpf, or 120 hpf. The embryos were anesthetized with 0.02% MS-222 solution, mounted in rows of 3% agarose plate, and photographed under the stereomicroscope SteREO Discovery V8 (Zeiss, Germany) equipped with DLT-Cam PRO 6.3 MP (Delta Optical, Warsaw, Poland) camera. The effects on the pigmentation were scored using ImageJ software version 1.51j8 (ImageJ, U. S. NIH, Bethesda, MD, USA). The results were presented as the area (µm^2^) covered by melanophores or iridophores.

### 2.4. RNA Extraction, Reverse Transcription, and qPCR Analysis

Gene expression analysis was performed in a 56 hpf wild-type Tuebingen zebrafish strain. Immediately following the TQR exposures, the embryos of the control and each experimental group were pooled (*n* = 30), frozen, and stored at −80 °C. Total RNA was extracted from pooled frozen embryos using a Total RNA Mini isolation kit (AA Biotechnology, Gdynia, Poland). All steps of isolation were assessed according to the respective manufacturer’s protocols. Homogenization required for RNA isolation was made using TissueLyser II (Qiagen, Dusseldorf, Germany). The cDNA samples were synthesized from respective high-quality matrix samples with equal RNA concentration for each sample using Maxima First Strand cDNA Synthesis Kit for RT-qPCR (Thermo Scientific, Waltham, MA, USA). All steps of reverse transcription were assessed according to the manufacturer’s protocols. qPCR was performed using SYBR Green in accordance with the manufacturer’s protocol (SYBR Select Master Mix, Applied Biosystems, Foster City, CA, USA) on 7500 Fast Real-Time PCR System instrument (Applied Biosystems, Foster City, CA, USA). A single PCR reaction included a 1 μL portion of the reverse transcription product. Oligonucleotide primers were designed to detect genes of chosen chromatophore differentiation markers, such as melanocyte inducing transcription factor (*mitf*), tyrosinase (*tyr*), tyrosinase-related protein 1 (*tyrp1*), GTP cyclohydrolase 2 (*gch2*), dopachrome tautomerase (*dct*), leukocyte tyrosine kinase (*ltk*), forkhead box D3 (*foxd3*), and purine nucleoside phosphorylase 4a (*pnp4a*), and two genes of zebrafish ABC transporter family—*abcb4* and *abcb5*. Initial validation of reference genes revealed that for the purpose of the study, elongation factor 1-alpha (*ef1-α*) showed the most efficient and equal expression among the samples. The details are listed in Table 1. The values of the expression of the studied genes were analyzed using the comparative Ct method and calculated in each group as a relative expression to *Ef1-α*. Each sample was analyzed in triplicate in three separate experiments. The qPCR protocol was used with a 7500 Fast Real-Time PCR System instrument (Applied Biosystems, Foster City, CA, USA), according to the previously established parameters [29].

### 2.5. Statistical Analysis

The statistical analysis was performed using GraphPad Prism, version 5.0 (GraphPad Software Inc., San Diego, CA, USA). Data with Gaussian assumption were analyzed using a one-way ANOVA test with Tukey multiple comparisons tests as a post hoc test or student’s t test. Data analyses not assuming Gaussian distribution were based on a Kruskal–Wallis test with Dunn’s multiple comparisons test as a post hoc test. The error bars represent means ± SEM. The significance level was set at *α* = 0.05 (95% confidence intervals).

## 3. Results

### 3.1. Effects of TQR on Size and Distribution of Melanophores

**Figure 1:**
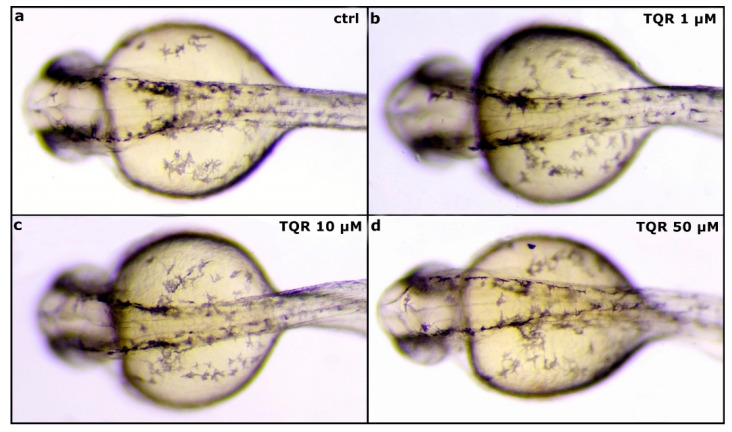
Zebrafish melanophore pigmentation following the exposure to tariquidar (TQR) at 30 h post fertilization (hpf). A set of photographs presenting a dorsal view of 30 hpf zebrafish embryo displaying effects of tariquidar (TQR) exposure on the melanophore pigmentation within the dorsal head in four experimental groups: (**a**) control, (**b**) exposed to TQR 1 µM, (**c**) exposed to TQR 10 µM, and (**d**) exposed to TQR 50 µM. The developing dark pigment pattern does not show visible differences between the groups.

**Figure 2:**
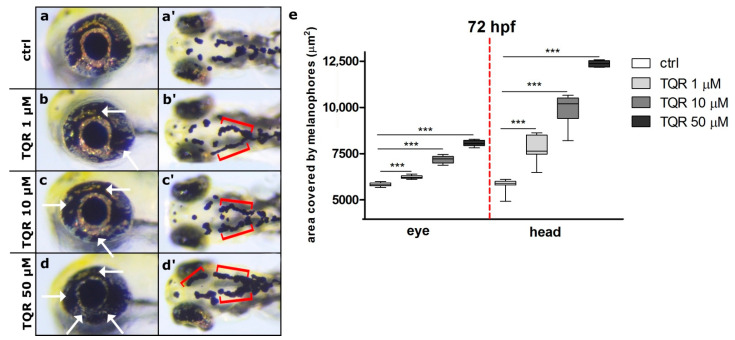
Dose-dependent increase in zebrafish melanophore pigmentation following the exposure to tariquidar (TQR) at 72 h post fertilization (hpf). A set of photographs presenting lateral and dorsal views of 72 hpf zebrafish larvae exhibiting effects of 68-h TQR exposure on the melanophore pigmentation within the eye and dorsal head in four experimental groups: (**a**,**a’**) control, (**b**,**b’**) exposed to TQR 1 µM (**c**,**c’**) exposed to TQR 10 µM, and (**d**,**d’**) exposed to TQR 50 µM. Control larvae presented normal melanophore distribution within both the eye and dorsal head (**a**,**a’**). Progressive hypermelanogenesis was observed as the concentration increased (**b**,**b’**,**c**,**c’**,**d**,**d’**). In the area of the eye, the melanophores started to occupy the area normally covered by the iridophores (**b**,**c**,**d**) (white arrows). Within the dorsal head in the control group, the melanophores were separated, while in the TQR exposed groups, they began to merge and form closely apposed groups (**b’**,**c’**,**d’**) (red buckle). (**e**) A graph presenting the area covered by melanophores (µm^2^) measured at 72 h post fertilization (hpf) within the eye and dorsal head (one-way ANOVA/Kruskal–Wallis, GraphPad Prism 5, *** *p* < 0.001).

**Figure 3:**
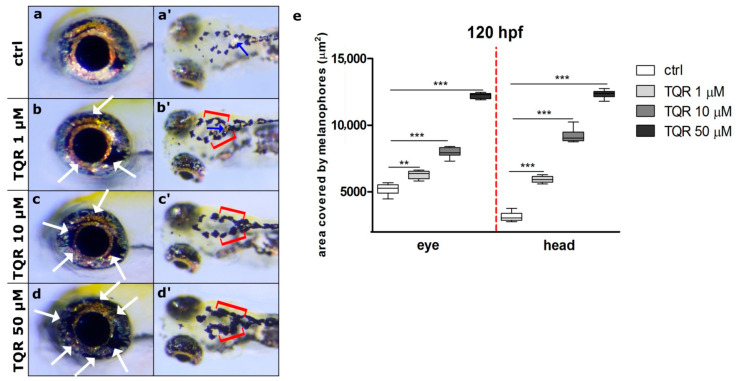
Dose-dependent increase in zebrafish melanophore pigmentation following exposure to tariquidar (TQR) at 120 h post fertilization (hpf). A set of photographs presenting lateral and dorsal views of 120 hpf zebrafish larvae displaying effects of 116 h TQR exposure on the melanophore pigmentation within the eye and dorsal head in four experimental groups: (**a**,**a’**) control, (**b**,**b’**) exposed to TQR 1 µM, (**c**,**c’**) exposed to TQR 10 µM, and (**d**,**d’**) exposed to TQR 50 µM. The control larvae presented normal melanophore distribution within both the eye and dorsal head (**a**,**a’**). At 120 hpf, the effects of TQR exposure were the continuation of those observed at 72 hpf; however, they were more intense. Progressive hypermelanogenesis was observed with increasing concentration (**b**,**b’**;**c**,**c’**;**d**,**d’**). In the area of the eye, the melanophores began to occupy the area normally covered by the iridophores (**b**,**c**,**d**) (white arrows). Within the dorsal head in the control group, the melanophores were separated and small, while in the TQR exposed groups they were expanded and started to merge creating form closely apposed groups (**b’**,**c’**,**d’**) (red buckle). Additionally, in the control (**a’**) and 1 µM TQR-exposed group (**a’**) in the central part of the head, the iridophores were visible (blue arrows), while in 10 µM TQR- and 50 µM TQR-exposed groups these cells were not found. (**e**) A graph presenting the area covered by melanophores (µm^2^) measured at 120 h post fertilization (hpf) within the eye and dorsal head (one-way ANOVA/Kruskal–Wallis, GraphPad Prism 5, *** *p* < 0.001, ** *p* < 0.01).

To demonstrate the properties of TQR as a melanogenic enhancer, first, 4 hpf embryos were exposed to three different TQR concentrations: 1 µM, 10 µM, and 50 µM. To establish the effects of TQR exposure during embryogenesis two distinct regions were chosen—the eye and dorsal head. During the first 48 h of development, no significant changes in the morphology and area covered by melanophores were observed between control and TQR-exposed groups (Figure 1). However, from 72 hpf, we observed progress, i.e., a dose-dependent increase in the area covered by melanophores (Figure 2). At 72 hpf, in the control group, the average area covered with melanophores within the dorsal head was 5820.7 ± 330.94 µm^2^ (Figure 2a’,e) and within the eye was 5829.5 ± 103.77 µm^2^ (Figure 2a,e). Exposure to 1 µM resulted in 1.4-fold increase in the area covered by melanophores within the dorsal head (8487.5 ± 456.0 µm^2^) (*p* < 0.001) (Figure 2b’,e) and 1.15-fold increase within the eye (6752.2 ± 139.20 µm^2^) (*p* < 0.001) (Figure 2b,e). In 10 µM-exposed group, we found 1.8-fold increase within the dorsal head (10,397.7 ± 425.41 µm^2^) (*p* < 0.001) (Figure 2c’,e) and 1.2-fold increase within the eye (7210.5 ± 210.42 µm^2^) (*p* < 0.001) (Figure 2c,e). The 50 µM exposure resulted in the highest increases at the level of 2.1-fold within the dorsal head (1236.7 ± 168.23 µm^2^) (*p* < 0.001) (Figure 2d,e) and 1.4-fold within the eye (8062.1 ± 150.28 µm^2^) (*p* < 0.001) (Figure 2d,e) compared to the control group. At 120 hpf, the dose-dependent trend persisted; however, the stimulatory melanogenic effect intensified in comparison to the results obtained at 72 hpf (Figure 3). In the control group, the average area covered with melanophores within the dorsal head was 3136.2 ± 342.21 m^2^ (Figure 3a’,e) and within the eye was 5168.2 ± 405.48 µm^2^ (Figure 3a,e). Within the dorsal head the exposure to 1 µM, 10 µM, and 50 µM resulted in, respectively, 1.9 (5921.9 ± 246.64 µm^2^) (*p* < 0.001) (Figure 3b’,e), 2.9 (9229.9 ± 554.44 µm^2^) (*p* < 0.001) (Figure 3c’,e) and 3.9 (12,331.1 ± 298.25 µm^2^) (*p* < 0.001) (Figure 3d’,e) fold increases in the area covered by melanophores compared to the control group. Within the eye the changes were slightly lower and represented 1.2 (6292.0 ± 285.31 µm^2^) (*p* < 0.01) (Figure 3b,e), 1.5 (7943.5 ± 345.70 µm^2^) (*p* < 0.001) (Figure 3c,e) and 2.3 (12,213.5 ± 199.22 µm^2^) (*p* < 0.001) (Figure 3d,e) fold increases in the respective concentrations of 1 µM, 10 µM, and 50 µM TQR. Moreover, especially within the dorsal head region, altered melanophore morphology was observed. They became expanded and formed closely apposed groups.

**Figure 4:**
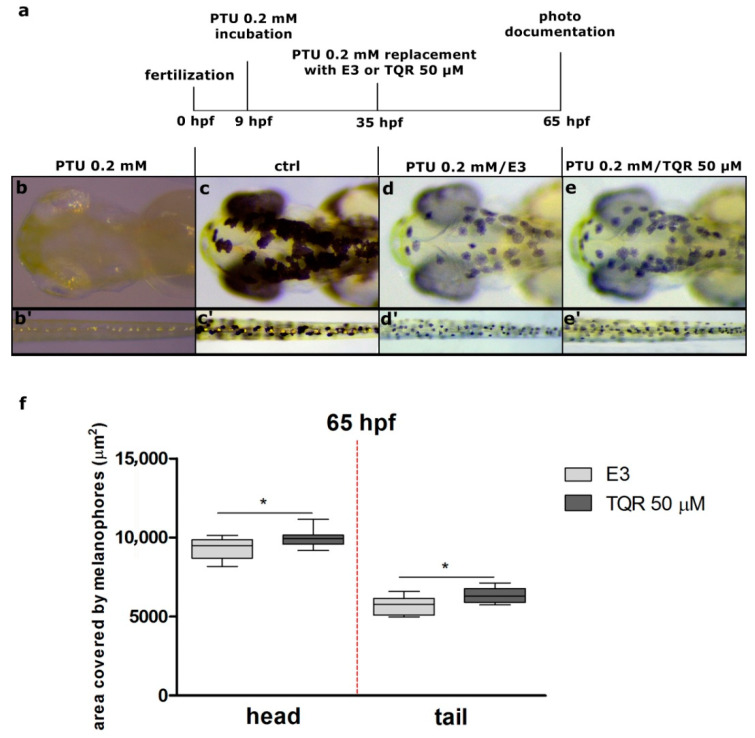
A set of pictures taken at 65 h post fertilization (hpf) showing the melanogenic stimulatory effect of 50 µM tariquidar (TQR) after 0.2 mM 1-phenyl-2-thiourea (PTU) exposure in larval zebrafish. (**a**) A schematic representation of the schedule of pigmentation rescue investigation. Nine-hpf embryos were pretreated with 0.2 mM PTU. At 35 hpf, all the embryos were washed and immersed in the E3 medium or 50 µM TQR solution. The incubation after medium replacement lasted until 60 hpf. Photographs present **(b,b’**) larvae treated with PTU from 9 to 65 hpf, (**c,c’**) control larvae incubated in E3, (**d,d’**) larvae treated with PTU from 9 to 35 hpf and then replaced with E3, and (**e,e’**) larvae treated with PTU from 9 to 35 hpf and then replaced with TQR. (**f**) A graph presenting the area covered by melanophores (µm^2^) measured at 65 h post fertilization (hpf) within the dorsal head and tail. Replacement with 50 µM TQR resulted in a significant increase in the area covered by melanophores (µm^2^), within both the dorsal head and tail, in the TQR-exposed group compared to that determined in the E3 medium treated group (student’s t-test, GraphPad Prism 5, * *p* < 0.05).

To confirm the stimulatory effect of TQR during melanogenesis, one more experiment was carried out. Firstly, 9 hpf embryos were exposed to 0.2 mM PTU to achieve no pigmented phenotype. After that, at 35 hpf, the PTU solution was replaced by E3 or 50 µM TQR, and the effects of this replacement were observed at 65 hpf. The results obtained showed that in the group in which PTU was replaced with 50 µM TQR the area covered with melanophores was moderately larger than in the group in which PTU was replaced with E3 (Figure 4). To determine the effects of TQR exposure after PTU treatment, two distinct regions were chosen—the tail and dorsal head. At 35 hpf, all embryos demonstrated transparent phenotype—neither melanophores nor iridophores were visible around the body. Moreover, 30 h after PTU replacement in the E3-exposed group, the average area covered with melanophores within the dorsal head was at the level of 9264.4 ± 694.0 µm^2^ (Figure 4d,f) and within tail was at the level of 5696.4 ± 546.76 µm^2^ (Figure 4d’,f). Replacement with 50 µM TQR resulted in slightly swifter melanophore renewal. In this group, the average area covered with melanophores within the dorsal head was at the level of 9942.0 ± 552.82 µm^2^ (*p* < 0.05) (Figure 4e,f) and within the tail was at the level of 6350.8 ± 470.75 µm^2^ (*p* < 0.05) (Figure 4e’,f).

### 3.2. Effects of TQR on Size and Distribution of Iridophores

**Figure 5:**
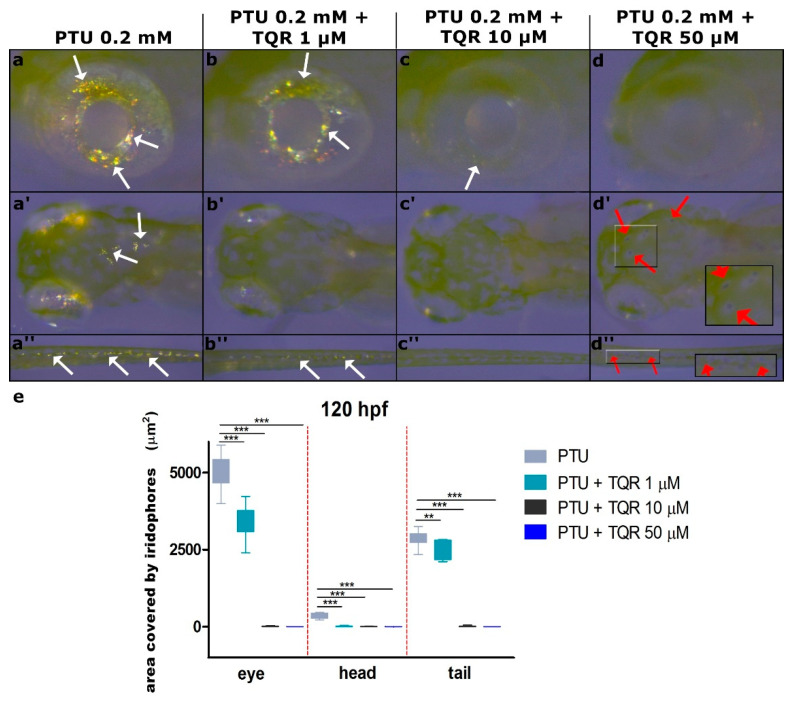
Dose-dependent decrease in zebrafish iridophore pigmentation following the exposure to the mixture of 0.2 mM 1-phenyl-2-thiourea (PTU) and increasing concentrations of tariquidar (TQR) at 120 h post fertilization (hpf). A set of photographs showing lateral and dorsal views of 120 hpf zebrafish larvae exhibiting effects of 68 h PTU + TQR exposure on the iridophore pigmentation within the eye, dorsal head, and tail in four experimental groups: (**a**,**a’**,**a”**) exposed to PTU 0.2 mM, (**b**,**b’**,**b”**) exposed to PTU 0.2 mM + TQR 1 µM (**c**,**c’**,**c”**) exposed to PTU 0.2 mM + TQR 10 µM, and (**d**,**d’**,**d”**) exposed to PTU 0.2 mM + TQR 50 µM. PTU treatment resulted in melanophore disappearance with no influence on the iridophores (**a**,**a’**,**a”**; white arrows). Co-treatment with 1 µM TQR caused a slight reduction in the area covered with the iridophores; however, they were still well visible, mostly within the eye and tail (**b**,**b”**) (white arrows). In the 10-µM TQR co-treated group, only single iridophores were visible within the eye (**c**) (white arrow), while in the area of the dorsal head and tail they disappeared entirely (**c’**,**c”**). The addition of 50 µM TQR to the PTU solution resulted in a complete depletion of the iridophores within all areas investigated (**d**,**d’**,**d”**); however, small and faint melanophores were detectable (red arrows) (**d’**,**d”**). (**e**) A graph presenting the area covered by iridophores (µm^2^) measured at 120 h post fertilization (hpf) within the eye, dorsal head, and tail (Kruskall–Wallis, GraphPad Prism 5, *** *p* < 0.001, ** *p* < 0.01).

To demonstrate the properties of TQR as an iridophore development silencer, 9 hpf embryos were exposed to three different TQR concentrations: 1 µM, 10 µM, and 50 µM, but each dose was additionally mixed with 0.2 mM PTU. This procedure was applied to assess the direct influence on iridophores excluding interferences with melanophores. This issue is more widely described in the Discussion Section. We observed that PTU treatment affects only melanophores, but not iridophore development. At 120 hpf in the PTU-exposed group, the average area covered with iridophores was respectively as follows: within the eye 5014.7 ± 551.16 µm^2^, within the dorsal head 359.2 ± 86.64 µm^2^, and within the tail 2856.7 ± 247.33 µm^2^ (Figure 5a,a’,a”,e). The addition of 1 µM TQR to the treatment solution resulted in a moderate reduction in the size of the area covered with iridophores. We found 1.5-fold decrease within the eye (3382.8 ± 524.13 µm^2^) (*p* < 0.01) (Figure 5b,e), 40-fold decrease within the dorsal head (9.2 ± 16.32 µm^2^) (*p* < 0.001) (Figure 5b’,e), and 1.15-fold decrease within the tail (2502.2 ± 290.08 µm^2^) (*p* < 0.01) (Figure 5b”,e). After the addition of 10 µM TQR to the treatment solution, we reported nearly complete extinction of the iridophores population. In most cases, the iridophores were undetectable, while in some individuals we observed the solitary cells, which covered the studied areas at the average level of 8.7 ± 12.45 µm^2^ within the eye (*p* < 0.001) (Figure 5c,e), 3.7 ± 6.49 µm^2^ within the dorsal head (*p* < 0.001) (Figure 5c’,e), and 8.8 ± 16.91 µm^2^ within the tail (*p* < 0.001) (Figure 5c”,e). In turn, the inclusion of 50 µM TQR to the treatment solution resulted in complete depletion of iridophores from all investigated areas (*p* < 0.001) (Figure 5d,d’,d”,e). However, we observed that in the 0.2 mM PTU + 50 µM TQR-exposed group, some melanophores began to appear (Figure 5d’,d”).

### 3.3. Effects of TQR on mRNA Expression Level of Chromatophore Differentiation Markers

**Figure 6:**
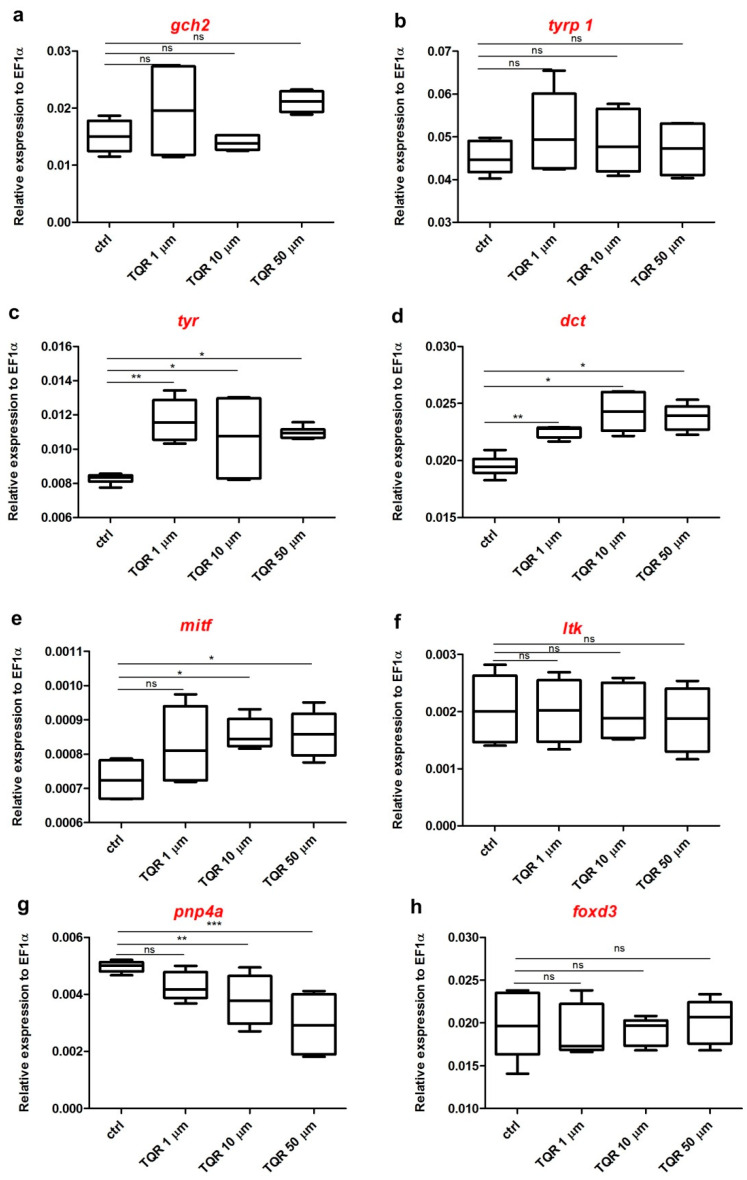
Expression profiles of chromatophore differentiation genes. The graphs show pooled data of the mRNA expression of (**a**) GTP cyclohydrolase 2 (*gch2*), (**b**) tyrosinase-related protein 1 (*tyrp1*), (**c**) tyrosinase (*tyr*), (**d**) dopachrome tautomerase (*dct*), (**e**) melanocyte inducing transcription factor (*mitf*), (**f**) leukocyte tyrosinase kinase (*ltk*), (**g**) purine nucleoside phosphorylase 4a (*pnp4a*), and (**h**) forkhead box D3(*foxd3*) from pooled 56-hour post-fertilization (hpf) wild-type zebrafish larvae (*n* = 30) in four experimental groups: 1) control, 2) exposed to TQR 1 µM, 3) exposed to TQR 10 µM, and 4) exposed to TQR 50 µM. Each group was covered by samples analyzed in triplicate in three separate experiments. Data in a figure represent the average of the three individual experiments. Gene expression values were normalized to housekeeping gene elongation factor 1-alpha *(ef1-α).* TQR had no statistically significant influence on *gch2* (**a**), *tyrp 1* (**b**), ltk (**f**), and foxd3 (**h**). *Tyr* (**c**) and *dct* (**d**) were significantly upregulated after the exposure to all studied TQR concentrations. *Mitf* (**e**) was not altered by TQR 1 µM; however, TQR in doses of 10 µM and 50 µM significantly increased the mRNA expression level. The *pnp4a* gene (**g**) was downregulated after the treatment with TQR, in dose-dependent manner (Kruskal–Wallis, GraphPad Prism 5, *** *p* < 0.001, ** *p* < 0.01, * *p* < 0.05, ns: not statistically significant differences (*p* > 0.05).

The effects of the exposure to 1 µM, 10 µM, and 50 µM TQR (lasted from 4 hpf until 56 hpf) on the mRNA level of various genes encoding chromatophore differentiation markers were determined by RT-qPCR (Figure 6). The analysis clearly showed that each dose of TQR had no statistically significant influence on *tyrp* and *gch2* and *foxd3* (*p* > 0.05) (Figure 6a,b,h). *Tyr* and *dct* were upregulated after the exposure to all studied TQR concentrations, however, not in an evident dose-dependent manner (*p* < 0.05–0.01) (Figure 6c,d). *Mitf* was not altered by 1 µM TQR (*p* > 0.05) (Figure 6e); however, after the treatment with higher TQR doses, a significant increase in mRNA expression level was found (*p* < 0.05) (Figure 6e). The mRNA level of *pnp4a* was reduced after the treatment with all studied TQR exposure doses in a dose-dependent manner (*p* < 0.01) (Figure 6g).

### 3.4. Effects of TQR on mRNA Expression Level of Abcb4 and Abcb5

**Figure 7:**
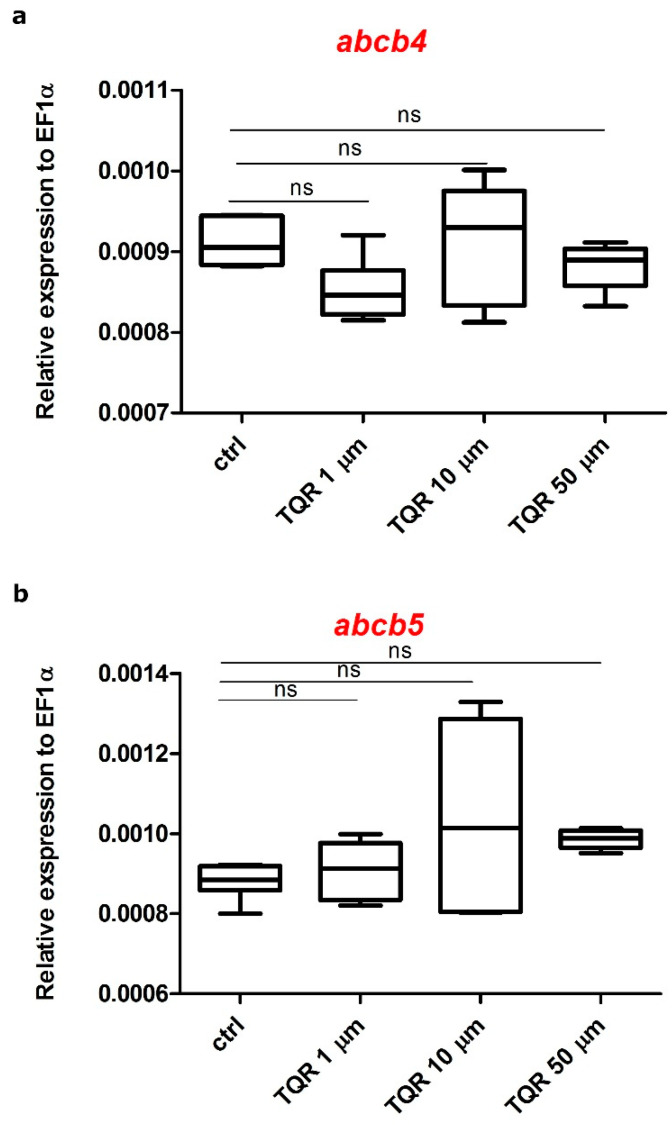
Expression profiles of zebrafish ATP-binding cassette (ABC) transporter family genes. The graphs show pooled data of the mRNA expression of (**a**) *abcb4* and (**b**) *abcb5* from pooled 56-hour post-fertilization (hpf) wild-type zebrafish larvae (*n* = 30) in four experimental groups: (1) control, (2) exposed to TQR 1 µM, (3) exposed to TQR 10 µM, and (4) exposed to TQR 50 µM. Each group was covered by samples analyzed in triplicate in three separate experiments. Data in the figure represent the average of the three individual experiments. Gene expression values were normalized to housekeeping gene *elongation factor 1-alpha (Ef1-α).* TQR had no statistically significant influence on both *abcb4* (**a**) and *abcb5* (**b**) (Kruskal–Wallis, GraphPad Prism 5, ns: not statistically significant differences (*p* > 0.05)).

The effects of the exposure to 1 µM, 10 µM, and 50 µM TQR (lasted from 4 hpf until 56 hpf) on the mRNA level of genes encoding Abcb4 and Abcb5 were determined by RT-qPCR (Figure 7). The expression of *abcb4* was not statistically significantly altered after the treatment with all studied TQR concentrations (*p* > 0.05) (Figure 7a). The analysis of the expression of *abcb5* gene revealed a slight but not statistically significant dose-dependent increase (*p* > 0.05) (Figure 7b).

## 4. Discussion

The present study provides, for the first time, clear evidence that the P-gp inhibition by TQR leads to distinct pigment pattern alteration in zebrafish. So far, many mammalian models were used to describe pigment regulatory compounds; however, recently zebrafish has emerged as a powerful and reliable model system in studies dealing with pigment development and diseases. As reviewed by Lister (2002) [31], before attention has been paid to the zebrafish pigment cells themselves, they served firstly as an instrumental model in the development of mutagenesis techniques and gene mapping in this organism. Today, zebrafish offers an excellent experimental system in the area of both the earliest events of pigment cell development and during adulthood. Moreover, due to the sequenced genome and incomparable with any other model organism amount of pigment mutants, zebrafish serves a lot of possibilities and genetic tools to study the molecular basis of pigment regulation and diseases. Furthermore, pigment cell abnormalities are medically relevant, because they are associated with neural crest originated disorders or those arising from dysfunction in RPE, and therefore, getting to know them can contribute to a better understanding of the disease pathogenesis.

As mentioned previously, in mammals, the pigment cell population consists of retinal pigment cells and melanocytes. Lower vertebrates, including fish and amphibians, possess additionally xanthophores and iridophores. In our study, we have focused on two types of zebrafish chromatophores—melanophores and iridophores. Our investigations clearly revealed that TQR exposure led to hypermelanogenesis in the dermis and RPE. The involvement of ABC transporters in melanogenesis has been partially described. Chen et al. (2005a, 2009) [27,32] and Heimerl et al. (2007) [33] identified the type-specific high expression of the *abcb5* gene in cells of melanocytic origin, such as melanocytes, RPE, and melanoma cells in human cell cultures. Interestingly, these authors have revealed that Abcb5 transcript level is relatively decreased in melanoma in comparison to normal pigment cells. Although in our study, the expression of *abcb5* remained unchanged after TQR exposure, the TQR as ABC efflux pump inhibited the protein function, presumably leading to similar effects, i.e., uncontrolled expansion of melanophores. We are far from concluding that after TQR exposure we obtained melanoma phenotype, but commonly, melanoma cells and melanophores after TQR exposure exhibit uncontrolled enlargement. The analysis of the *abcb4* gene in human cell cultures with cells of melanocytic origin showed its unchanged expression between cell types, which was lower than that of the *abcb5* gene, suggesting that *abcb5* is a pigment cell-specific marker [33]. Zebrafish possesses only two P-glycoprotein genes annotated as *abcb1* orthologs, *abcb4* and *abcb5* [28]. It was found that Abcb4, but not Abcb5, protein acts as an active barrier against chemical uptake and confers the resistance to studied Abcb1 substrates [28]. Interestingly, the expression of zebrafish *abcb5* was found in epidermal cells [34]. Therefore, it has been suggested that Abcb5 protein plays a role similar to that found in mammals and is responsible for regulating membrane potential and cell fusion of skin progenitor cells [32,35]. In normal pigment-producing cells, the ABC transporter system, primarily Abcb5, regulates and maintains melanocyte homeostasis by trapping cytotoxic melanin intermediates into subcellular organelles such as endosomes, lysosomes, and melanosomes [32]. P-glycoprotein system inhibition by TQR disturbed this homeostasis and led to an altered phenotype of melanocytes, manifested by their expansion and tendency to form closely apposed groups. However, it is still under consideration, whether melanocyte fusion was the effect of their rapid growth and spontaneous merger or Abcb5 glycoprotein blockade, as this phenomenon was previously described by Frank et al. (2003) in human epidermal melanocyte (HEM) cell culture [35].

The majority of melanogenic regulatory compounds studied in zebrafish are defined as tyrosinase stimulators or inhibitors [14]. Since TQR has been never tested as a melanogenic regulatory agent, we aimed at investigating whether it acts due to its stimulatory potential on tyrosinase activity. Gene expression analysis revealed the upregulation of *tyr* after TQR exposure. The product of the *tyr* gene is an enzyme called tyrosinase, which is located in melanocytes and is involved in the first step of melanin production. Extensive studies on tyrosinase gene expression in zebrafish embryos described the pattern of its transcription, confirming *tyr* activity both in RPE and neural crest-derived melanocytes [8]. The present results suggest that presumably TQR simultaneously affects the regulation of pigment-related genes both in RPE and skin melanocytes, which is manifested by visible hyperpigmentation in these two structures. However, some studies demonstrated opposite results, where some of the tyrosinase inhibitors constrained skin depigmentation only without influencing RPE [14], suggesting that the process of RPE pigmentation is somehow different from that of body pigmentation in zebrafish. Moreover, we observed a slight competitive effect of the highest TQR dose in comparison to that triggered by PTU. PTU as a strong tyrosinase silencer inhibits melanogenesis, but the co-exposure with 50 µM TQR resulted in the emergence of small dermis melanophores.

TQR exposure (10 and 50 µM) increased the expression of the *mitf* gene. *Mitf* is able to respond to multiple signaling pathways and is responsible for helping the control of various genes involved in melanocyte development, survival, differentiation, and functioning [36]. Initially, *mitf* was shown to play its key role in the direct transcriptional control of *tyr*, *tyrp1,* and *dct* genes, which are associated with the process of melanocyte differentiation [36,37]. Currently, much more *mitf* target genes are known, among which there are genes involved in, e.g., cell cycle arrest (*p16*, *p21*) and survival (*bcl-2*, *hif1α*, *c-met*) [36,37]. In our study, we tested genes involved in differentiation—*tyr*, *tyrp1*, and *dct*. *Dct* and *tyr* (described in more detail above) were significantly upregulated after TQR exposure, while *tyrp1* expression remained unchanged. However, based on this data it is difficult to establish whether this is a direct effect of TQR exposure or *mitf* overexpression upshot.

Next to the observation of TQR impact on melanophores, we also found its influence on iridophores. In wild-type larvae, we observed the opposite effect after TQR exposure —it stimulated the melanophores and inhibited the iridophores. Since the interaction between pigment cells was revealed to play a key role in pigment pattern formation [38,39,40,41], we aimed to check whether the iridophore lost after TQR exposure was a result of direct TQR action or increased melanogenesis. For this purpose, we co-treated the larvae with increasing doses of TQR and 0.2 mM PTU to get rid of melanophores. Surprisingly, we observed the direct effect of TQR on iridophore survival. The exposure to 10 and 50 µM of TQR led to their almost total disappearance. Moreover, *pnp4a*, which is considered as a new marker of early iridoblast development and differentiation [10,12], was significantly downregulated in TQR treated animals. This shed new light on the role of TQR in pigmentation in zebrafish. Several mutations are known to be responsible for a reduction of iridophores [6,42,43]. *Shady* (encoding leukocyte tyrosine kinase) mutants lack iridophores both in larvae and in adults, whereas in *rose* (encoding endothelin receptor b1a) or *kar* (encoding endothelin-converting enzyme), mutants only the adult pattern is affected [13,44,45]. Importantly, all those iridophores lacking mutants also demonstrate deficits in melanophore number and altered stripe formation in adult age. In our study, we investigated *ltk* gene expression, because as mentioned, this mutation alters both the larval and adult stage. *Ltk* remained unchanged after TQR exposure pointing to the involvement of other gene-regulating iridophore fate. In our study, we observed that both pigment cells—iridophores and melanophores—are altered, which suggests that melanophores and iridophores develop from a common precursor. This may also imply that their faith is regulated by one factor or there is some kind of interplay between the core gene regulatory network governing this process. We tested this hypothesis by determining the gene expression profile of *foxd3*, *pnp4a,* and the described earlier *mitfa*. Curran et al. [10] proposed the model in which a cell can either develop directly into one of the two chromatophores or it may pass through a bi-potent stage before acquiring its ultimate fate. This fate is regulated by *foxd3*, which reappears in approximately half of bi-potent precursors at 24 hpf, resulting in repression of *mitfa* and activation of *pnp4a* [10]. We hypothesized that simultaneous *mitfa* upregulation and *pnp4a* downregulation may arise from altered *foxd3* expression. Surprisingly, *foxd3* remained unchanged after TQR exposure, both at 56 hpf and 24 hpf (data not shown), indicating that TQR exposure influences *mitfa* and *pnp4a* independently, or other mechanisms are involved. Moreover, taking into consideration well-described interactions between zebrafish chromatophores (mostly studied in adult zebrafish), our data exclude the possibility of the classic influence of one cell type on the other. It has been shown that iridophores promote and sustain melanophores and that mutants lacking iridophores are also characterized by a decreased number of melanophores [46]. Furthermore, iridophores attract xanthophores, whereas xanthophores repel melanophores [46]. Therefore, the direct interaction between the studied pigment cells does not appear to be present. Although xanthophores were not the object of our study, Figure 5 demonstrates that the yellowish layer on the head is present in unchanged form in all groups investigated, hence excluding the direct influence of these cells on the fate of melanophores and iridophores.

## 5. Conclusions

The present study has demonstrated, for the first time, the influence of the third generation non-competitive P-gp inhibitor, TQR, on pigment pattern regulation in zebrafish. We have focused on the phenotype effects and supported the findings obtained with some relevant molecular analyses. The final results suggest that pigment pattern regulation by TQR is multidirectional and would imply that those processes are presumably cell lineage dependent. They also suggest that TQR should be considered as a factor playing an important regulatory role in pigment cell development or survival, and it cannot be excluded that this interesting property may have a wider biological context.

## Figures and Tables

**Table 1:** Primers used in the study.

Gene	Forward 5′-3′	Reverse 5′-3′	Source/Accession No.
*mitf*	AGGACCTTGAAAACCGACAG	GTGGATGGGATAAGGGAAAG	[30]/NM_001178049
*tyr*	GATCCAGGTCAGCGGTTTGT	ACCGATGCGATTATTCCTGCT	[30]/NM_131013.3
*tyrp1*	GGCCACCTATCAGAAACGCT	AGTGTATGCCCGAGTTGGC	[30]/NM_001002749.2
*gch2*	GTTGTCATTGAAGCAGCTCACA	TCTGAACACACCCAGCATCG	NM_131667.1
*dct*	CAGCTTCAGGAATGCACTGG	GCTGGTCCCATTGAGGAACT	[30]/NM_131555.2
*abcb4*	TACTGATGATGCTTGGCTTAATC	TCTCTGGAAAGGTGAAGTTAGG	[28]/JQ014001
*abcb5*	CGCTGGTCATTCTGGCTGTC	CTCCTCTGCTACCGCTCCAG	[28]/JQ014002
*ltk*	GGTTTTGACAGCGACGGTTC	TGCCCGTTCTCCATCCGATA	NM_001006660.1
*pnp4a*	GGTTTTGACAGCGACGGTTC	CGGTGCTGTACTCATTCCAACT	NM_001002102.1
*foxd3*	ATCAAATCCGAGCCGTCCAG	CGGGTTAAGGACAGGGACTG	NM_131290.2
*ef1-α*	CTGGAGGCCAGCTCAAACAT	ATCAAGAAGAGTAGTACCGCTAGCATTAC	NM_131263.1

## Data Availability

Data available on request from the authors.

## List of Abbreviations

Hpf	hours post fertilization
Dpf	days post fertilization
TQR	tariquidar (TQR; *N*-[2-[[[4-[2-(3,4-Dihydro-6,7-dimethoxy-2(1*H*)-isoquinolinyl)ethyl]phenyl]amino]carbonyl]-4,5-dimethoxyphenyl]-3-quinolinecarboxamide)
RPE	retinal pigment epithelium
gch2	cyclohydrolase 2
tyrp1	tyrosinase-related protein 1
tyr	tyrosinase
dct	dopachrome tautomerase
mitf	melanocyte inducing transcription factor
pnp4a	purine nucleoside phosphorylase 4a
foxd3	forkhead box D3
ef1-α	elongation factor 1-alpha
